# Homozygous deep intronic variant in SNX14 cause autosomal recessive Spinocerebellar ataxia 20: a case report

**DOI:** 10.3389/fgene.2023.1197681

**Published:** 2023-07-06

**Authors:** Olga Levchenko, Alexandra Filatova, Irina Mishina, Aleksey Antonenko, Mikhail Skoblov

**Affiliations:** ^1^ Research Centre for Medical Genetics, Moscow, Russia; ^2^ Evogen LLC, Moscow, Russia

**Keywords:** ataxia, intellectual and developmental disabilities, SCAR20, deep intronic variants, SNX14 gene, whole genome sequencing

## Abstract

Autosomal recessive spinocerebellar ataxia type 20, SCAR20 (MIM: 616354) is a rare syndromic form of hereditary ataxias. It characterized by the presence of progressive ataxia, intellectual developmental disorder, autism and dysmorphic features. The disease caused by biallelic variants in *SNX14* gene that lead to loss of protein function. Typically, these variants result in the formation of a premature stop codon, a shift in the reading frame or a variant in canonical splicing sites, as well as gross rearrangements. Here we present the first case of a deep intronic variant c.462-589A>G in *SNX14* identified in two sisters with SCAR20 from a consanguineous family. This variant resulted in the inclusion of a pseudo-exon 82 nucleotides long and the formation of a premature stop codon, leading to the production of a truncated protein (NP_722523.1:p.Asp155Valfs*8). Following an extensive diagnostic search, the diagnosis was confirmed using trio whole genome sequencing. This case contributes to expanding the spectrum of potential genetic variants associated with SCAR20.

## 1 Introduction

The hereditary progressive ataxias comprise genetic disorders that affect the cerebellum and its connections (Didonna and Opal, 2016). Autosomal dominant spinocerebellar ataxia (SCA) has the largest number of forms, followed by autosomal recessive (SCAR) and the smallest group is X-linked ataxia (SCAX) (Klockgether et al., 2019). Phenotypic series of autosomal recessive spinocerebellar ataxia (MIM: PS213200) includes 29 genes in which different types of variants can lead to disease. Homozygous or compound heterozygous variants in *SNX14* lead to Autosomal Recessive Spinocerebellar Ataxia 20. This is the ultra-rare disease, with only 28 pathogenic and likely pathogenic variants described in the ClinVar database, in more than 36 patients from 19 families. Most of known causative variants assume the loss of protein functions (LoF) (Bryant et al., 2018). Typically, SCAR20 appears by intellectual disability, ataxia, coarse facies, and cerebellar atrophy. Unlike many other hereditary ataxias, SCAR20 has distinctive facial features like broad face, fullness of the upper eyelid, broad nasal base and slight underdevelopment of the alae, broad and long philtrum, thick lower lip vermillion (Thomas et al., 2015).

Often the diagnosis of SCAR could be challenging due to the high genetic heterogeneity with phenotypic similarity (Arias, 2019). To address these challenges, specialized tests such as neuroimaging, neurophysiological assessments, and genetic testing are commonly used. Genetic testing can involve searching for expanded short tandem repeats, target sequencing panels, WES, or WGS (Galatolo et al., 2018). The search for expansions is the most effective diagnostic method for dominant ataxias, especially in familial cases with late onset (Chen et al., 2018), while NGS is often used for SCARs (Renaud et al., 2017). If we analyze different variants of NGS with each other, then more often in the literature there are data on more effective diagnostics using whole exome sequencing (WES) (up to 41%) compared to sequencing a gene panels (up to 18%), even a large one, including 90–10 genes (Willemsen and Kleefstra, 2014; Shakya et al., 2019). With careful clinical diagnosis in some large centers, the efficiency of panel sequencing can reach 30% (Riso et al., 2021). However, this result should be considered rather as an exception to the rule, besides the rate of discovery of new genes makes panel sequencing less efficient (Galatolo et al., 2018). Thus, WES is the optimal diagnostic method for SCARs (Shakya et al., 2019).

In complex cases, the most effective is whole genome sequencing (WGS), as it allows to identify not only exon variants, but also variants located in introns, which can be regulatory or affect splicing (Belkadi et al., 2015). Intronic variants that could alternate splicing are increasingly recognized as responsible for monogenic disorders. About 15%–50% of all monogenic disease-causing mutations affect pre-mRNA splicing. WGS has resulted in the identification of an increasing number of pathogenic variants located deep within introns (i.e., more than 100 base pairs away from exon–intron boundaries). These findings are fostering a new era of research focused on understanding how variation in deep intronic sequence affects pre-mRNA splicing and contributes to disease phenotypes (Vaz-Drago et al., 2017).

In this report, we describe a consanguineous family with two affected sisters, who have a homozygous variant located deep within an intronic region of the *SNX14* gene, 589 base pairs away from the exon-intron junction. We demonstrated the molecular pathogenic mechanism underlying the formation of a novel donor splicing site within intron 5, resulting in the inclusion of a pseudo exon in RNA, leading to a frameshift.

## 2 Materials and methods

### 2.1 Clinical report

**FIGURE 1:**
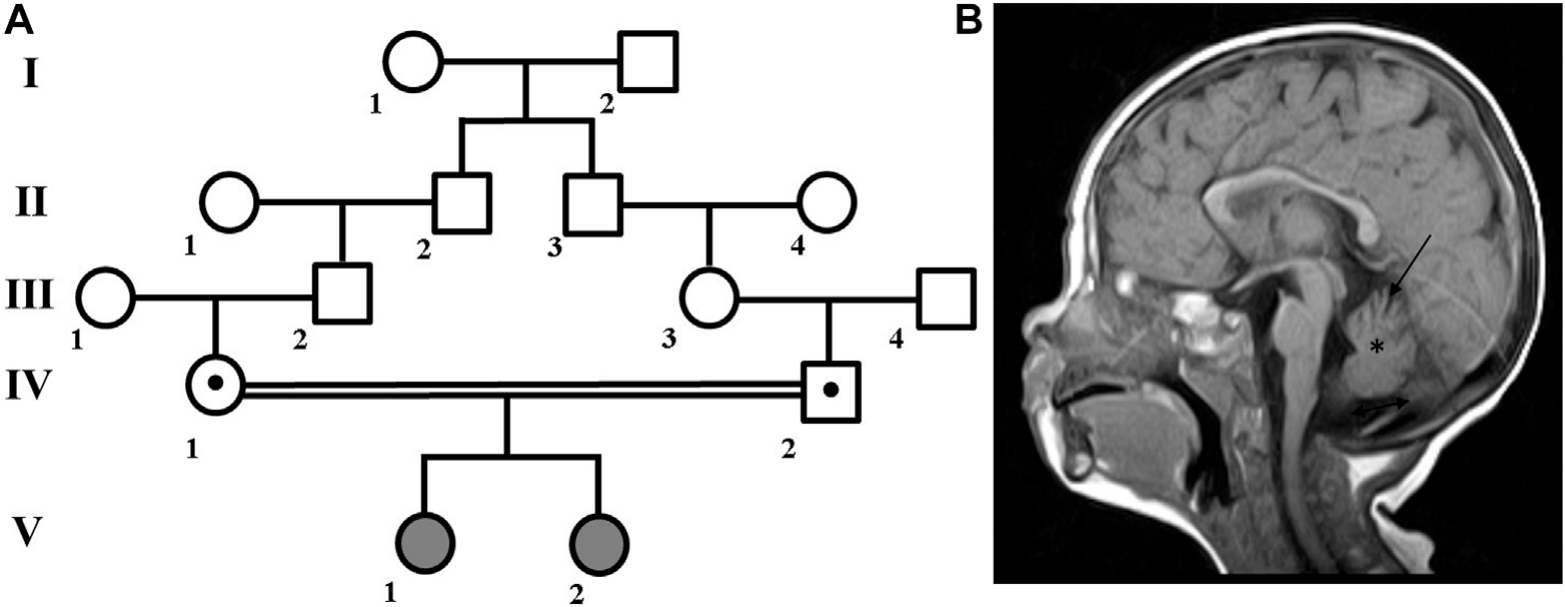
Family pedigree and brain MRI scan. **(A)** Family pedigree. **(B)** Brain MRI scan from patient V:1 showed cerebellar atrophy (arrow), reduced volume of the cerebellum (asterisk), widening of the posterior cranial fossa (double arrow), periventricular leukopathy (not shown).

A family with two affected children (Figure 1A) was examined at the Research Centre for Medical Genetics. The couple, who were Russian and belonged to the Northeast Caucasian ethnic group Avars, were closely related as second cousins. The first pregnancy ended in stillbirth at 40 weeks of gestation, medical records were not available.

Patient V:1 is the first female child. Her mother experienced both toxicosis and anemia during the second pregnancy. At birth, she weighed 3,750 g, measured 52 cm in length, and received a good Apgar score of 8/8. However, she had neonatal jaundice for up to 2 months and was hypotonic. As she developed, she experienced delays such as holding her head up at 2 months, sitting at 11 months, walking with ataxia at 1 year and 7 months, and saying her first words at 2 years old. She is currently 13 years old, and while her growth and weight parameters have been normal, her occipitofrontal circumference (OFC) has measured 56 cm (+1.88 SD), which is above average.

In addition, she has hypertelorism, epicanthus, dynamic ataxia, wide terminal phalanges of the thumbs and toes. She is able to speak a few words and eat with a spoon, but is not able to dress herself or perform self-care tasks. In terms of neurological status, patient exhibits spontaneous nystagmus, high pharyngeal and palatine reflexes, muscle hypertrophy of the limbs, reduced tendon reflexes, and no elicited knee and Achilles reflexes. Babinski’s reflex is present on both sides. MRI revealed cerebellar atrophy and periventricular leukopathy (Figure 1B).

Patient V:2 is a 7-year-old female who exhibits clinical features similar to her older sister. She was born during the third pregnancy, which was also complicated by toxicosis and anemia. At birth, she weighed 3,260 g, measured 52 cm in length, and received a good Apgar score of 9/9. However, within the first 2 weeks of life, her bilirubin levels increased to 205 μg/L.

Since birth, Patient V:2 has experienced decreased reflexes, and her psychomotor development has been significantly delayed. She held her head up at 3 months, sat at 10 months, and walked unsteadily at 1 year and 2 months. Physical examination has revealed hypertelorism, epicanthus, high palate, wide umbilical ring, transverse palmar crease, and dynamic ataxia. In the neurological status, she has high tendon reflexes, and Babinski’s reflex is present on both sides. Nerve conduction study (NCS) results showed normal nerve conduction and amplitudes muscle responses.

### 2.2 Molecular investigation

Patient V:1 underwent testing for short tandem repeats in several genes, including *CACNA1A*, *ATXN1, ATXN2, ATXN3, ATXN7, ATXN8, PPP2R2B, FXN*, and *ATN1*, which yielded negative results. Her karyotype was determined to be 46,XX. Furthermore, no variants were identified in her mitochondrial genome, WES, or chromosomal microarray (CMA) analyses.

### 2.3 Genome sequencing and variants calling

Trio whole genome sequencing (trio WGS) was performed in the case V:1. DNA was extracted from blood using a QIAamp DNA Blood Mini Kit (Qiagen). DNA purity was assessed by measuring DNA absorbance with NanoDrop (Thermo Fisher Scientific) at both 260/280 nm and 230/260 nm. DNA concentration was measured by Qubit fluorometer (Thermo Fisher Scientific) and a total of 2 µL of DNA, regardless of concentration, was then electrophoresed on an high-resolution capillary electrophoresis to determine the level of DNA fragmentation. Library preparation (PCR-Free) using MGI platforms then proceeded as protocols. Paired-end sequencing (2 х 150) was performed on the DNBSEQ-T7 (MGI). The data processing was carried out using “NGSData-Genome” program (Beskorovainy N.S. Program “NGSData”//Certificate of NGSData-Genome”//Certificate of State Registration of Computer Programs No. 2021662119.2021.) The reads were aligned to the reference genome hg19 using bwa v.0.7.17-r1188. Variants calling was performed with strelka2 v.2.9.10 and gatk v.4 algorithms. Colling of copy number variations–cnvkit 0.9.9; structural variants–manta v.1.6.0; tandem repeats–ExpansionHunterDenovo 0.9. Variant annotation–SnpEff v5.0, annovar v.2017, vep v.104.3. Splice predictors–dbNSFP v.4, SPiP v.2.1, mmsplice v.2.3, spliceai v.1.3.1, spidex v.1.

### 2.4 Segregation studies

The identified variants were confirmed by Sanger sequencing for the parents and affected sisters, using the ABI PRISM Big Dye Terminator (v 3.1) Cycle Sequencing Kit (Applied Biosystems, Foster City, CA, United States) on the ABI3130xl Genetic Analyzer (Applied Biosystems, Foster City, CA, United States).

### 2.5 Transcript analysis

Total RNA from peripheral blood mononuclear cells (PBMCs) of one affected individual, both parents and three unrelated healthy controls were extracted using ExtractRNA reagent (Evrogen, Russia) according to the manufacturer’s recommendations. RNA samples were treated with DNAseI (Thermo Fisher Scientific, United States) and reverse transcribed using 5X RT MasMIX −30100 (Dialat ltd, Russia). A region harboring *SNX14* (NM_153816.6) exons 3 to 7 was amplified using primers *SNX14*-ex3F (5’-TCA​TAG​CTG​TGC​TGT​TTG​TGG-3’) and *SNX14*-ex7R (5’-TGC​TTC​ATT​GCT​GCT​TTT​AAT-3’). PCR products were separated on a 2% agarose gel. Each DNA band was gel purified using Cleanup Standard kit (Evrogen, Russia) and further sequenced as described above.

## 3 Results

**FIGURE 2:**
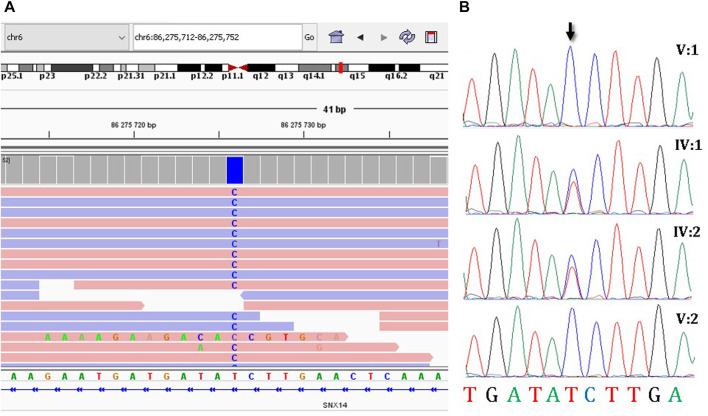
The molecular results of the diagnostic testing. **(A)** Homozygous deep-intronic variant c.462-589A>G is visualized using the Integrative Genomics Viewer (IGV), **(B)** Sanger sequencing results of segregation analysis.

Due to the presence of two affected family members with a similar clinical features, a hereditary pathology was highly to be assumed and it was decided to continue molecular genetic diagnosis. Since WES and CMA were inconclusive, a trio-WGS was performed on patient V:1 and her parents to search for variants in non-coding regions of the genome. The average coverage of the patient’s genome was 29.2, coverage of target loci greater than 10x was 96.1%. Next, we searched for rare intronic variants that were predicted by SpliceAI (Jaganathan et al., 2019) as highly likely to affect splicing (Δ score > 0.6) and their frequency was less than 0.05%. This search resulted in the identification of 24 variants that met the criteria. In four cases, the genes in which variants were identified were associated with a hereditary disease. Only the variant NM_153816.6:c.462-589A>G in *SNX14* (ClinVar: SCV003852744) was identified (Figure 2A) in the homozygous state and matched the search criteria. Autosomal recessive spinocerebellar ataxia, type 20, has been associated with homozygous and compound heterozygous variants in this gene, which fully corresponds to the clinical picture of the sisters. Same as in the described patients, the sisters had an intellectual development disorder, motor delay, relative macrocephaly, coarse face, cerebellar hypotrophy, and severe ataxia. The variant was absent in the GnomAD database v2.1.1., v3.1.2., and in 219 Russian genomes. Sanger sequencing confirmed that this variant was heterozygous in the healthy parents and homozygous in the affected sisters (Figure 2B). The variant was classified as variant of unknown significance (PM2) according to ACMG criteria (Richards et al., 2015).

**FIGURE 3:**
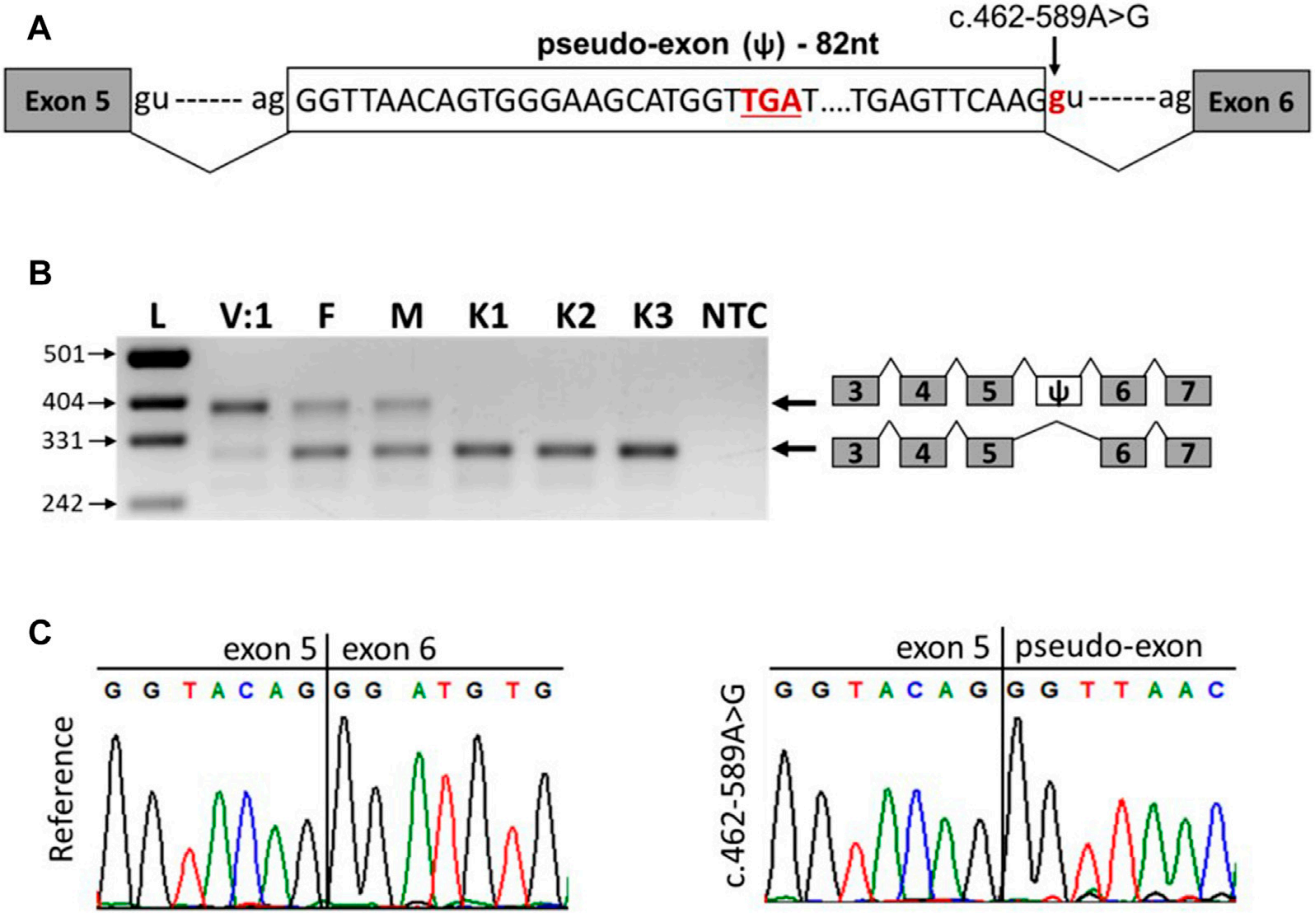
The c.462-589A>G variant leads to the inclusion of pseudo-exon between *SNX14* exons 5 and 6. **(A)** Scheme of the observed pseudo-exon, the premature stop codon is underlined. **(B)** RT-PCR analysis of *SNX14* mRNA from PBMCs of patient V:1 (homozygous carrier of the variant), her parents (heterozygous carriers of the variant) and three unrelated controls. **(C)** Sanger sequencing results of reference and aberrant (c.462-589A>G) PCR products.

The c.462-589A>G variant is a deep intronic variant located in intron 5 of the *SNX14* gene. SpliceAI analysis predicted that the variant created a new donor splicing site (with a delta score of 0.63) and activated a cryptic acceptor splice site 82 bp downstream of the variant. Together, these can lead to the inclusion of a pseudo-exon into the *SNX14* mRNA (Figure 3A). To confirm this hypothesis, we performed reverse transcription PCR (RT-PCR) analysis of the total RNA isolated from peripheral blood mononuclear cells (PBMCs) of the patient V:1, her parents and three unrelated healthy controls (Figures 3B,C).

The RNA analysis revealed that the c.462-589A>G variant leads to the formation of a new donor splicing site within intron 5, resulting in a pseudo-exon inclusion between exons 5 and 6 of the *SNX14* gene (Figures 3B,C). Such aberrant transcript was detected in the patient and her parents and was not detected in healthy controls (Figure 3B). The pseudo-exon is 82 nucleotides long and its inclusion leads to the appearance of a premature stop codon with the formation of a truncated protein (NP_722523.1:p.Asp155Valfs*8) (Figure 3A). After analysis the variant was reclassified as likely pathogenic (PS3, PM2) according to ACMG criteria (Richards et al., 2015). Interestingly, in the homozygous carrier of the variant (patient V:1), we observed a residual amount of wild-type transcript (Figure 3B).

## 4 Discussion

The clinical presentations of hereditary ataxias may overlap with other neurological conditions, making it difficult to establish an accurate diagnosis. The choice of a methodological approach to diagnostics, as well as its efficiency can vary depending on several factors such as the specific type of ataxia, clinical characteristics of the patient, and the age of onset (Arias, 2019). Despite the diversity and high quality of modern approaches to diagnostics, some cases may still go undiagnosed, particularly in individuals with atypical presentations or rare forms of ataxia or rare genetic variants out of exons. In such situations, WGS is a powerful tool as it can detect copy number variations, analyze non-coding regions of the genome that may contain crucial regulatory elements or cryptic exon sites, and has the potential to reveal rare genetic variants that might otherwise be missed by other diagnostic methods (Meienberg et al., 2016). Human genomes typically contain on average at least 10 pseudo-exon activation events. More often pseudo-exon activation events happen in 5' donor splice sites than in 3' acceptor splice sites (Sakaguchi and Suyama, 2021). Pseudoexon inclusions have been described as the cause of many diseases, such as cystic fibrosis (Shibata et al., 2020) or core myopathy (Rendu et al., 2013).

In this report, we describe two sisters from a consanguineous family with SCAR20 caused by the inclusion of a pseudoexon due to a deep intronic variant (NM_153816.6:c.462-589A>G) in the *SNX14* gene. The variant resulted in the formation of a new donor splicing site within intron 5, leading to pseudo exon inclusion, a frameshift, and a premature stop-codon in position 155. The heterozygous carriers showed the ratio of the upper and lower bend on electrophoresis (Figure 3B) is not equals, according to densitometry suggesting that the allele carrying the pathogenic variant is partially subject to nonsense-mediated decay and partially results in a truncated protein. Furthermore, the patient’s sample showed residual amount of wild-type transcript. It may be due to the strength of pseudoexon splicing sites is equal to the strength of the wild-type sites, and in some cases, during competition for the spliceosome, the inclusion of the pseudoexone does not occurs. For some genes it is known dose-dependency in phenotypic severity (Nakano et al., 2020), and minor amount of normal protein may lead to a milder phenotype compared to complete loss of function (Kondratyeva et al., 2021). However, despite the presence of pathogenic variants leading to stop codons after position 155 in other patients, their phenotype did not significantly differ from our patients, except for the presence of neonatal hyperbilirubinemia in both sisters. No other genetic variants in WGS have been identified to explain hyperbilirubinemia. The younger sister had increased tendon reflexes, which is not typical for this disease, but in rare cases are described (Thomas et al., 2015). Perhaps in the future, the sisters will have a milder clinical picture. However, we will not be able to distinguish between variable expressivity and the effect of gene dose due to the lack of specific markers. For the same reason, we did not measure the exact amount of the wild-type transcript. Thus, we decided to consider c.462-589A>G in *SNX14* as a disease-causing variant.

## 5 Conclusion

In this study we broaden the *SNX14* mutational spectrum with deep-intronic variant. Describing two patients from consanguine family with SCAR20. In our case WGS analysis was only possible diagnostic test that could reveal cause of disease. Due to genetic heterogeneity of ataxias the WGS has a significant advantage over WES for diagnostic.

## Data Availability

The data presented in the study are deposited in the ClinVar repository, accession number SCV003852744.
